# Chromosomal Integration of the Klebsiella pneumoniae Carbapenemase Gene, *bla*_KPC_, in Klebsiella Species Is Elusive but Not Rare

**DOI:** 10.1128/AAC.01823-16

**Published:** 2017-02-23

**Authors:** Amy J. Mathers, Nicole Stoesser, Weidong Chai, Joanne Carroll, Katie Barry, Anita Cherunvanky, Robert Sebra, Andrew Kasarskis, Tim E. Peto, A. Sarah Walker, Costi D. Sifri, Derrick W. Crook, Anna E. Sheppard

**Affiliations:** a Division of Infectious Diseases and International Health, Department of Medicine, University of Virginia Health System, Charlottesville, Virginia, USA; b Clinical Microbiology, Department of Pathology, University of Virginia Health System, Charlottesville, Virginia, USA; c Modernizing Medical Microbiology Consortium, Nuffield Department of Clinical Medicine, University of Oxford, Oxford, United Kingdom; d Icahn Institute and Department of Genetics and Genomic Sciences, Icahn School of Medicine, Mount Sinai, New York, New York, USA; e Office of Hospital Epidemiology, University of Virginia Health System, Charlottesville, Virginia, USA

**Keywords:** carbapenemase, KPC, Klebsiella, Klebsiella pneumoniae carbapenemase, antibiotic resistance, chromosomal, plasmid analysis, plasmids, transposons, whole-genome sequencing

## Abstract

Carbapenemase genes in Enterobacteriaceae are mostly described as being plasmid associated. However, the genetic context of carbapenemase genes is not always confirmed in epidemiological surveys, and the frequency of their chromosomal integration therefore is unknown. A previously sequenced collection of *bla*_KPC_-positive Enterobacteriaceae from a single U.S. institution (2007 to 2012; *n* = 281 isolates from 182 patients) was analyzed to identify chromosomal insertions of Tn*4401*, the transposon most frequently harboring *bla*_KPC_. Using a combination of short- and long-read sequencing, we confirmed five independent chromosomal integration events from 6/182 (3%) patients, corresponding to 15/281 (5%) isolates. Three patients had isolates identified by perirectal screening, and three had infections which were all successfully treated. When a single copy of *bla*_KPC_ was in the chromosome, one or both of the phenotypic carbapenemase tests were negative. All chromosomally integrated *bla*_KPC_ genes were from Klebsiella spp., predominantly K. pneumoniae clonal group 258 (CG258), even though these represented only a small proportion of the isolates. Integration occurred via IS*15*-ΔI-mediated transposition of a larger, composite region encompassing Tn*4401* at one locus of chromosomal integration, seen in the same strain (K. pneumoniae ST340) in two patients. In summary, we identified five independent chromosomal integrations of *bla*_KPC_ in a large outbreak, demonstrating that this is not a rare event. *bla*_KPC_ was more frequently integrated into the chromosome of epidemic CG258 K. pneumoniae lineages (ST11, ST258, and ST340) and was more difficult to detect by routine phenotypic methods in this context. The presence of chromosomally integrated *bla*_KPC_ within successful, globally disseminated K. pneumoniae strains therefore is likely underestimated.

## INTRODUCTION

Carbapenem resistance in Enterobacteriaceae has become a major clinical challenge (1). Within this bacterial family, carbapenemase genes are largely located on plasmids cocirculating with various strains (2). Plasmid DNA may act as a temporary “lending library,” enabling genes of importance to survive various selective pressures (3). However, *in vitro* and modeling data suggest that once a gene is incorporated chromosomally, it will be maintained through replication without selective pressure, and gene loss from the bacterial population becomes less likely (4–6).

The Klebsiella pneumoniae carbapenemase gene (*bla*_KPC_) is maintained within the self-mobilizing 10-kb transposon Tn*4401* (7). The frequency of transposition *in vitro* is relatively high (4.4 × 10^−6^/recipient cell) and without site specificity, but the rate of movement outside laboratory settings is largely unknown (8). Nevertheless, Tn*4401* has been described in several different genetic environments (9, 10).

Historically, most descriptions of *bla*_KPC_ have been in a globally successful lineage of K. pneumoniae, namely, multilocus sequence type 258 (ST258) and the associated clonal group 258 (CG258), which includes ST11, ST258, and ST340 (11). CG258 isolates are widespread, albeit concentrated in geographic hotspots, with the *bla*_KPC_ gene being plasmid associated in the majority of reports (9). Interestingly, the earliest observed chromosomal Tn*4401* integration events have been sporadic and in non-Klebsiella spp.: Pseudomonas aeruginosa in 2006 (12), Raoultella spp. in 2008 (13) and Acinetobacter baumannii in 2009 (14). More recently, chromosomal integration among K. pneumoniae ST258 (*n* = 4) isolates has been described in the United States by three separate groups (15–17).

The rate of chromosomal integration within clinical settings for many genetic elements is largely unknown. Chromosomal integration of *bla*_KPC_ may be underreported, as its investigation often requires detailed genetic analysis, and if a plasmid copy is present then a chromosomal copy may be overlooked. Even high-resolution genetic methods, such as whole-genome sequencing (WGS) using short-read technologies, can be confounded by the presence of multiple copies of Tn*4401* and/or repetitive flanking sequences, which limit the ability to accurately reconstruct the genetic context(s) of *bla*_KPC_ (10, 18). Here, we use a combination of short- and long-read WGS to investigate chromosomal integration of *bla*_KPC_ among 281 isolates (62 distinct strains) of *bla*_KPC_-positive Enterobacteriaceae isolated from 182 patients in a single hospital.

## RESULTS

### Identification of chromosomal integrations of *bla*_KPC_.

There were 62 distinct strains (from 13 species) among 281 sequenced K. pneumoniae carbapenemase (KPC)-Enterobacteriaceae isolates derived from 182 infected/colonized patients (18). Using *de novo* assemblies to identify isolates where Tn*4401* was adjacent to known chromosomal sequences, 44% (123/281) of isolates, from 82/182 (45%) patients, were evaluable (Table 1). In 7/123 evaluable isolates, from 3/82 patients, Tn*4401* flanking sequence showed homology with chromosomal reference sequences, indicating a likely chromosomal location for *bla*_KPC_. Three different chromosomal loci were identified, one for each patient, demonstrating three distinct Tn*4401* chromosomal integration events. Isolates from two of the patients were K. pneumoniae (ST258 and ST340, with earliest isolates CAV1453 and CAV1417, respectively), and one was K. oxytoca (CAV1752).

**TABLE 1:** Species breakdown for isolates that were evaluable by the *de novo* assembly approach and chromosomal references used for mapping

Species	No. (%) of evaluable isolates for *de novo* assembly approach	Reference strain for mapping approach	Reference accession no.
Citrobacter amalonaticus	0/2 (0)	CAV1321^*a*^ (Citrobacter freundii)	CP011612
Citrobacter freundii	17/30 (57)	CAV1321	CP011612
Enterobacter aerogenes	3/4 (75)	EA1509E	NC_020181.1
Enterobacter asburiae	1/1 (100)	NCTC 9394^*b*^ (Enterobacter cloacae)	NC_021046.1
Enterobacter cloacae	18/96 (19)	NCTC 9394	NC_021046.1
Escherichia coli	2/2 (100)	DH10B	NC_010473.1
Klebsiella pneumoniae	42/94 (45)	MGH78578	CP000647.1
Klebsiella oxytoca	33/35 (94)	E718	NC_018106.1
Kluyvera intermedia	3/7 (43)	CAV1151	CP011602
Proteus mirabilis	0/1 (0)	HI4320	NC_010554.1
Raoultella ornithinolytica	0/1 (0)	B6	NC_021066.1
Serratia marcescens	4/5 (80)	WW4	NC_020211.1
Other (unknown)	0/3 (0)	NCTC 9394^*a*^ (E. cloacae)	NC_021046.1
Total	123/281 (44)		

a No species-specific reference available.

b E. cloacae reference used for simplicity, as E. asburiae is part of the E. cloacae complex.

As the *de novo* assembly approach was only able to assess 44% of isolates for possible chromosomal integrations, we also used a mapping approach, which is unaffected by multiple Tn*4401* copies. Reassuringly, all seven isolates identified by the *de novo* assembly approach described above were also identified by this method as having a likely chromosomal integration of Tn*4401*. In addition, the mapping approach identified a further 11 isolates as having putative chromosomal integrations, all of which were unevaluable by the *de novo* assembly approach. Six of these were K. pneumoniae ST11 isolates from a single patient (the earliest isolate was CAV1351). Three were K. pneumoniae ST340 isolates from two additional patients (earliest isolates were CAV1217 and CAV1518). One was a non-CG258 K. pneumoniae isolate (ST244; CAV1042) from an additional patient, and one was a K. oxytoca isolate (CAV1755) unrelated to CAV1752 (>40,000 single-nucleotide variant [SNV] differences), also from an additional patient.

To confirm the chromosomal locations of Tn*4401*, we used long-read sequencing. For 6/8 patients identified above, the earliest isolate from each was sequenced using PacBio. In 5/6 cases, the presence of Tn*4401* on the chromosome was confirmed, and in one case (CAV1042) it was refuted. For one of the two remaining patients (the earliest isolate was CAV1351), we did not perform additional PacBio sequencing, as a subsequent isolate from the same patient (CAV1392; 1 SNV difference from CAV1351) was previously PacBio sequenced as part of another study and shown to contain two copies of Tn*4401*, one chromosomal and one plasmid (18). CAV1518 from the final patient was not PacBio sequenced, as it was demonstrated using other methods (see below) to be a false positive from the mapping approach, since Tn*4401* was not present on the chromosome.

Altogether, we found five distinct loci of chromosomal integration of *bla*_KPC_ verified by long-read sequencing from six patients, with two additional patients having isolates that were falsely identified by the mapping approach (Table 2). All chromosomal integrations were in Klebsiella spp., mostly K. pneumoniae CG258, even though CG258 represented only a small proportion of the outbreak (4/18 versus 2/164 non-CG258 patients, *P* = 0.0009; 13/34 versus 2/247 isolates, *P* < 0.0001; Fisher's exact test).

**TABLE 2:** Epidemiology, susceptibilities, and genomic description for the earliest isolate from each patient with a possible chromosomal integration of *bla*_KPC_ as identified by mapping

Chromosomal integration status	Isolate	Species	MLST	Clinical microbiology finding					Tn*4401* coverage (relative to chromosome)	qRT-PCR mean fold change	Tn*4401* isoform and SNV variant (18)	Porin gene status^*a*^		No. and location of Tn*4401* loci
				Ertapenem VITEK 2 (μg/ml)/disk (mm)	Meropenem VITEK 2 (μg/ml)	Meropenem broth dilution (μg/ml)	Indirect carbapenemase test	Modified Hodge test				*ompK35*	*ompK36*	
No; suspected by mapping, rejected by long-read sequencing	CAV1042	K. pneumoniae	ST244	≥8/NA^*b*^	≥16	8	Positive	Positive	4.9	6.69	b-1	Intact	Intact	2 plasmid
Yes; suspected by mapping, confirmed by long-read sequencing of CAV1392	CAV1351	K. pneumoniae	ST11	≥8/15	2	8	Initially negative (subsequent subculture weak positive)	Initially negative (subsequent subculture weak positive)	3.1	1.89	b-2	Frameshift at aa 170	Intact	1 chromosomal, 1 plasmid
Yes; suspected by assembly and mapping, confirmed by long-read sequencing	CAV1453	K. pneumoniae	ST258	≥8/12	≥16	16	Negative	Weak positive (slight indent)	1.1	1.31	a-1	Frameshift at aa 42	Intact	1 chromosomal
Yes; suspected by mapping, confirmed by long-read sequencing	CAV1217	K. pneumoniae	ST340	≤0.5/26	≤0.25	0.5	Negative	Negative	2.3	0.88	b-6	Intact	Intact	1 chromosomal, 1 plasmid
Yes; suspected by assembly and mapping, confirmed by long-read sequencing	CAV1417	K. pneumoniae	ST340	≥8/no zone	8	8	Negative	Negative	1.0	0.77	b-7	54-kb insertion at aa 21	54-kb insertion at aa 112	1 chromosomal
No; suspected by mapping, rejected by TSD sequence examination	CAV1518	K. pneumoniae	ST340	4/17	≥16	8	Positive	Positive	4.5	3.55	b-2	Intact	Intact	Unknown
Yes; suspected by assembly and mapping, confirmed by long-read sequencing	CAV1752	K. oxytoca		≤0.5/24	≤0.25	2	Positive	Negative	1.0	0.27	b-1	10-kb insertion at aa 29	Intact	1 chromosomal
Yes; suspected by mapping, confirmed by long-read sequencing	CAV1755	K. oxytoca		≥8/15	≥16	32	Positive	Positive	8.5	4.77	b-1	Intact	Intact	1 chromosomal, 2 plasmid

a Intact indicates that the open reading frame is maintained with respect to the reference sequence.

b NA, not applicable.

### Mechanism of chromosomal integration in K. pneumoniae ST340.

**FIG 1:**
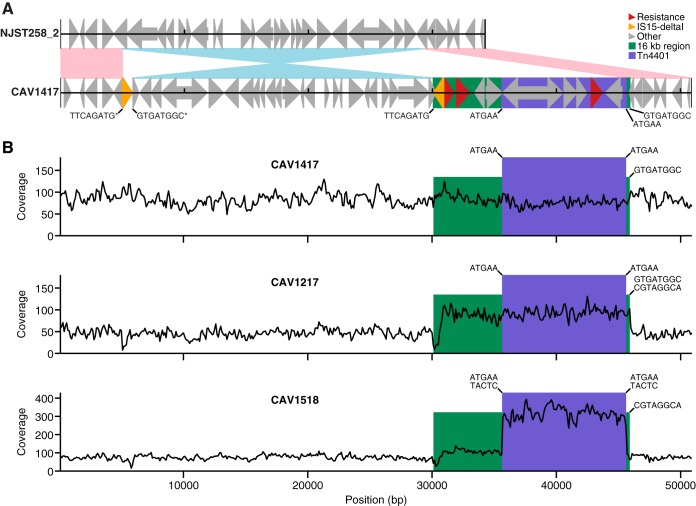
Chromosomal integration of Tn*4401* in Klebsiella pneumoniae ST340 isolates. (A) Alignment of ∼50-kb region of the CAV1417 chromosome with the homologous region of the NJST258_2 reference genome. Pink and blue shading indicate high sequence similarity (>99.8%) in the same or opposite orientations, respectively. Flanking sequences for mobile elements present in the complete (PacBio) CAV1417 genome are indicated (an asterisk indicates that the sequence shown has been reverse complemented). (B) Coverage of Illumina reads for CAV1417, CAV1217, or CAV1518 mapped to the complete CAV1417 genome across the region shown in panel A. Flanking sequences for Tn*4401* and the right side of the 16-kb region were determined from the mapped Illumina reads.

There were three patients with K. pneumoniae ST340 isolates where the mapping approach identified Tn*4401* as having a possible chromosomal location (CAV1417, CAV1217, and CAV1518) (Table 2). To assess the molecular basis of chromosomal integration, the chromosomal region encompassing Tn*4401* in the closed PacBio assembly of the ST340 CAV1417 isolate was aligned to the NJST258_2 reference (GenBank accession number CP006918), which belongs to the closely related ST258. Relative to the reference, CAV1417 had a 24-kb inversion and a 16-kb insertion, which included Tn*4401* and several other resistance genes (Fig. 1A).

At one end of the 16-kb region was an IS*15*-ΔI element, highly genetically similar (3 nucleotide differences) to IS*26*, both of which undergo replicative transposition with 8-bp target site duplication (TSD) (19). IS*26* has been shown to undergo frequent intramolecular transposition, which can result in inversion of the sequence between the original and duplicated elements. This process disrupts the pairing of TSD sequences, which can be used to trace the history of transposition events (20).

The other end of the 16-kb region terminated in a sequence with high similarity to the 14-bp IS*15*-ΔI inverted repeat (IR) sequence (IS*15*-ΔI, GGCACTGTTGCAAA; other, GGCTTTGTTGAATA; 10/14 nucleotide identities), suggesting that this acted as a cryptic recognition site mediating transposition of the entire 16-kb region, similar to a composite transposon. The 8-bp flanking sequences of the 16-kb region were identical to the 8-bp flanking sequences of a neighboring IS*15*-ΔI element located at the other end of the 24-kb inversion relative to NJST258_2 (Fig. 1A). This indicates that the 16-kb region initially integrated into the chromosome by intermolecular transposition, with an 8-bp TSD (GTGATGGC). Subsequently, IS*15*-ΔI underwent intramolecular transposition, resulting in a 24-kb inversion, with duplication of a second 8-bp sequence (TTCAGATG), with one copy flanking each of the duplicated elements.

In CAV1217, long-read sequencing demonstrated two copies of Tn*4401*, one chromosomal and one plasmid. For the chromosomal copy, the 8-bp sequence adjacent to the non-IS*15*-ΔI side of the 16-kb region (Fig. 1, right side) was identical to CAV1417 (GTGATGGC), while the plasmid copy had a different 8-bp flanking sequence (CGTAGGCA).

In CAV1518, the above-described signature of chromosomal integration (GTGATGGC sequence adjacent to the 16-kb region) is not present (Fig. 1B). Instead, the flanking sequence is identical to the plasmid copy in CAV1217 (CGTAGGCA). Therefore, the 16-kb region is most likely plasmid located in CAV1518, indicating that this isolate was a false positive from the mapping method.

### Clinical microbiologic characteristics.

Susceptibility results and phenotypic testing for these isolates varied substantially (Table 2). K. pneumoniae CAV1453, CAV1351, and CAV1417 were high-level carbapenem resistant but with weak to negative carbapenemase phenotypes. All of these isolates had a sequence disruption in major K. pneumoniae porin channels (*ompK35* and/or *ompK36*) (Table 2). CAV1453 had only a single Tn*4401* copy in the chromosome; however, it was Tn*4401*a, which generally results in increased KPC expression compared to Tn*4401*b, as was seen here with a higher level of expression compared to the other isolates with a single copy of Tn*4401*b in the chromosome (21, 22). It was negative by indirect carbapenemase testing (23) but weakly positive by the modified Hodge test. CAV1417 had a single chromosomal copy of Tn*4401*b, and phenotypic testing for carbapenemase activity was consistently negative, with low-level *bla*_KPC_ expression by quantitative PCR. Despite negative carbapenemase production by phenotypic tests, CAV1417 was highly carbapenem resistant and had disruptions in both *ompK35* and *ompK36*. The functional consequences of these disruptions have not been confirmed, but loss of function of these genes is expected to increase carbapenem resistance above that conferred by KPC alone (11, 21, 22, 24).

K. oxytoca isolate CAV1755 was carbapenem resistant and phenotypically positive by carbapenemase testing. This was also true for the K. pneumoniae isolate CAV1042, which was ultimately rejected as having a chromosomal copy. Both of these isolates had multiple loci of Tn*4401* integration (including a plasmid) with increased coverage of Tn*4401b* relative to the chromosome and also demonstrated increased *bla*_KPC_ expression and intact porin channel sequences.

CAV1217 was not noted to be *bla*_KPC_ positive until the patient was found to be colonized through perirectal screening (25) with a K. pneumoniae isolate with a weakly positive indirect carbapenemase test. This prompted further testing of the phenotypically carbapenem-susceptible K. pneumoniae urine isolate (CAV1217), which was then found to be *bla*_KPC_ PCR positive. This isolate had no disruption in the *ompK35* and *ompK36* coding regions and had two copies of Tn*4401* (one plasmid and one chromosomal) but with relatively low coverage.

K. oxytoca CAV1752, which had a single chromosomal copy of Tn*4401*b with low-level expression by quantitative PCR (qPCR), was detected on perirectal screening, again with conflicting phenotypic results (Table 2) (23). On automated susceptibility testing (VITEK 2), it was predicted to be susceptible to ceftriaxone and cefepime (MIC of ≤1 μg/ml) and carbapenems, with an aztreonam MIC of 4 μg/ml. Analysis of porin genes demonstrated an insertion disrupting *ompK35*.

### Clinical course and epidemiology.

All six patients confirmed to have chromosomal integration of *bla*_KPC_ had multiple risk factors and prolonged hospital exposure either within our health system or at outside hospitals prior to transfer (Table 3). Several patients with extensive outside health care exposure had isolates identified relatively early during hospitalization, raising the possibility of isolates circulating elsewhere in the region at outside hospitals. Interestingly, the patient with CAV1518 (the ST430 K. pneumoniae without chromosomal integration but with an ancestor common to CAV1217 and CAV1417) presented at an outside hospital prior to exposure to our health system, consistent with persistence of this lineage elsewhere in the region.

**TABLE 3:** Clinical and epidemiological characteristics

Isolate	Source and date (mo/yr) of earliest isolate with chromosomal integration	Epidemiology (local and outside-hospital exposure)	Comorbidity(ies)	Treatment characteristic					Infection with KPC isolate with chromosomal integration^*a*^	Treatment	30-day outcome
				Prior broad-spectrum intravenous antimicrobial in last 90 days	Ventilator for >48 h (during that hospital stay)	Indwelling central venous catheter	Foley catheter	In ICU for >48 h			
CAV1351	Sputum, 2/2011	Hospital day 5; extensive outside-hospital exposure	Persistent respiratory failure in setting of influenza pneumonia	+	+	+	+	+	Yes, VAP	Meropenem, tigecycline, amikacin	Microbiologic and clinical cure
CAV1453	Perirectal, 10/2011	Hospital day 3; extensive outside-hospital exposure	Cerebral palsy, chronic ventilator dependence	+	+	+	+	+	No	NA^*b*^	NA
CAV1217	Urine, 8/2010	Hospital day 3 (had similar K. pneumoniae in urine on admission which was not retained); extensive outside-hospital exposure	Diabetes mellitus, recurrent UTIs	+	−	−	+	−	Yes, cUTI	Foley catheter exchange with meropenem for 72 h changed to tigecycline for lack of improvement (persistent symptoms and unchanged bacterial burden)	Microbiologic and clinical cure
CAV1417	Urine, 5/2011	Hospital day 36	Chronic ventilator, neuromuscular weakness	+	+	+	+	+	Yes, VAP	Meropenem and colistin	Microbiologic and clinical cure
CAV1752	Perirectal, 12/2012	Hospital day 100; first *bla*_KPC_-positive isolate was K. pneumoniae identified 3 mo earlier during prior hospitalization	Orthotopic liver transplant	+	+	+	+	+	No	NA	NA
CAV1755	Abdominal drainage, 12/2012	Hospital day 26; first *bla*_KPC_-positive isolate was E. aerogenes, identified 10 mo earlier during prior hospitalization	End-stage renal disease, diabetes mellitus, complicated intra-abdominal infection	+	+	+	+	+	No	NA (had been previously successfully treated for E. aerogenes intra-abdominal infection	NA

a cUTI, complicated urinary tract infection; VAP, ventilator-associated pneumonia.

b NA, not applicable.

There were three patients who had infections (two with ventilator-associated pneumonia and one complicated urinary tract infection) with a K. pneumoniae isolate with chromosomal integration of *bla*_KPC_. The outcomes were complete microbiologic resolution and clinical cure at 30 days without any patients having known relapse within our health system.

The two patients with distinct strains of K. oxytoca (CAV1752 and CAV1755) did not demonstrate signs of infection with these isolates, thus the clinical consequences remain unclear. Interestingly, both patients had been colonized months before with other *bla*_KPC_-positive Enterobacteriaceae. As new acquisition at our institution is relatively rare and there were no other patients with similar isolates, this finding suggests separate within-patient horizontal transfer and chromosomal integration of Tn*4401*.

## DISCUSSION

We demonstrate, for the first time, multiple instances of chromosomal integration of Tn*4401/bla*_KPC_ within a single-center outbreak. Although this is not the first description of chromosomal integration of Tn*4401*, the frequency of integration in a clinical setting is largely unknown and likely overlooked. With a focused effort on identifying all instances of chromosomal integration, this event does not appear to be rare. In addition, we found that phenotypic carbapenemase testing was variable in these contexts, and *bla*_KPC_ could be missed by nonmolecular methods.

Illumina sequencing is increasingly used to undertake epidemiological investigation but is limited in investigating multicopy regions, which are of particular relevance to assessing resistance genes and mobile genetic elements. We utilized two bioinformatic approaches to characterize the genetic contexts of Tn*4401*. First, we used a *de novo* assembly approach, which identified three distinct chromosomal integrations. These were all verified by PacBio sequencing, demonstrating the robustness of this method. However, the method cannot identify chromosomal integration if multiple copies of Tn*4401* are present or if Tn*4401* is flanked by repetitive sequences, and only 44% of isolates were evaluable using this method. Second, we used a mapping approach which was able to assess all isolates but which identified several false positives. Therefore, results from the mapping approach on its own should be interpreted with caution, and a combination of methods may be most appropriate for other studies investigating sequence-based identification of resistance gene integration into the chromosome.

*bla*_KPC_ chromosomal integration was overrepresented among CG258 strains in our outbreak, suggesting that it is more widespread in high-risk clones such as K. pneumoniae ST258. This phenomenon has been seen in the high-risk Escherichia coli ST-131 clone, with chromosomal integration of the CTX-M beta-lactamase gene followed by vertical transmission (26, 27). It remains unclear if there is a propensity for chromosomal integration among certain K. pneumoniae strains or, more likely, whether integration is associated with the extended timespan over which these strains have been associated with Tn*4401*, representing greater opportunity for random integration.

**FIG 2:**
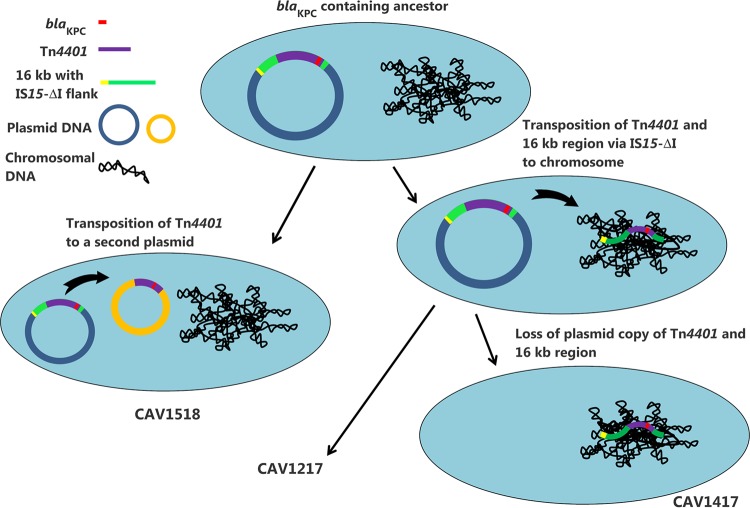
Schematic showing the presumed sequence of events affecting *bla*_KPC_ in Klebsiella pneumoniae ST340 isolates.

Within K. pneumoniae ST340, we identified three patients with putative chromosomal integrations of Tn*4401*, confirmed by PacBio sequencing in two isolates, CAV1217 and CAV1417. Comparison of the chromosomal region surrounding Tn*4401* in CAV1417 to a related strain lacking Tn*4401*, combined with the analysis of TSD sequences, allowed us to reconstruct the history of transposition events. This revealed that chromosomal integration of Tn*4401* occurred via IS*15*-ΔI-mediated transposition of a 16-kb region encompassing Tn*4401*. Further analysis of TSD sequences from Illumina data for CAV1518 revealed that the 16-kb region was not integrated into the chromosome in this isolate but rather shared flanking sequence with the plasmid copy from CAV1217. Taken together, this indicates that Tn*4401* most likely was acquired once in this lineage, in a common ancestor of all three isolates, with chromosomal integration occurring after the divergence of CAV1518 (in the ancestor of CAV1217 and CAV1417) and subsequent loss of the plasmid copy in CAV1417 (Fig. 2).

Another important feature of this study was the variability in phenotypic carbapenem resistance. A prior evaluation of phenotypic carbapenemase testing in Enterobacteriaceae from our institution (May 2010 to December 2011), using an ertapenem MIC of ≥1 μg/ml by VITEK 2 for inclusion, demonstrated that the indirect carbapenemase test had 90% sensitivity for detecting *bla*_KPC_-producing isolates (23). Isolates with chromosomally integrated *bla*_KPC_ accounted for 3 of the 5 false-negative *bla*_KPC_ Enterobacteriaceae identified in that study (total of 56 isolates tested). β-Lactamase carriage on high-copy-number plasmids compared to low-copy-number plasmids is known to alter the degree of resistance seen phenotypically (22, 28). Here, we found preliminary evidence that a single chromosomal copy of Tn*4401* was associated with a more subtle carbapenemase phenotype and lower expression of *bla*_KPC_. For example, CAV1752 would not have been detected with our current methods. This also could contribute to the misclassification of these isolates in a typical clinical microbiology laboratory, where the cost of molecular screening is often prohibitive (25).The differential impact on expression of resistance of *bla*_KPC_ located in the chromosome alone remains an interesting finding but will require additional *in vitro* studies to confirm this effect.

The impact of porin channel alterations may also affect the degree of *in vitro* resistance, highlighted here when comparing the related isolates CAV1417 and CAV1217. Although CAV1217 had two Tn*4401* copies and CAV1417 a single copy, CAV1417 had disruptions in both porin channel genes and was more phenotypically resistant. Nevertheless, porin channel loss does not always result in high resistance (e.g., CAV1752 has a disruption in *ompK35* but remained carbapenem susceptible), and the prediction of phenotype from genotype in these cases is challenging.

Our small number of cases is insufficient to reliably assess the clinical impact of low MICs and subtle phenotypes associated with chromosomal integration. However, these cases are not notably distinct from other descriptions of infection with *bla*_KPC_-positive K. pneumoniae, with all infected patients ultimately responding to therapy (29). Interestingly, CAV1217 was phenotypically susceptible; however, despite 48 h of meropenem treatment with an initial Foley exchange, the patient had persistent symptoms and no change in bacterial burden but was ultimately treated successfully with tigecycline (Table 3).

This work also highlights that the frequency of chromosomal integration of genes of drug resistance is largely unknown and would be missed by most methods. Even with WGS, sophisticated focused analysis was required to identify chromosomal integration when a plasmid copy was also present. Currently, *bla*_KPC_ is carried within a replicative transposon, and although the *in vitro* transposition frequency of this element is relatively high, the real-world rate is unknown (8). Although the frequency of chromosomal integration cannot be fully assessed without additional study, this remains an intriguing preliminary finding from prospectively collected isolates in a clinical setting.

In summary, we present five independent examples of chromosomal integration of Tn*4401-bla*_KPC_ in Klebsiella spp. from six patients among a comprehensively characterized outbreak, indicating that chromosomal integration is not an infrequent event. We also demonstrate that chromosomal integration often may be overlooked even with WGS, especially when multiple Tn*4401* copies are present. In the clinical microbiology laboratory, phenotypic tests for carbapenem resistance/carbapenemase production were variable and could also miss these cases. Thus, chromosomal integration of *bla*_KPC_ was relatively common, was difficult to detect, and may facilitate stable inheritance of *bla*_KPC_, representing an evolutionary adaptation with potential implications for surveillance and treatment.

## MATERIALS AND METHODS

### Isolate collection, characterization, and selection for whole-genome sequencing.

We previously described short-read (Illumina) sequencing of 281 *bla*_KPC_-positive Enterobacteriaceae isolates, from 182 patients, collected between August 2007 and December 2012 in the University of Virginia Health System (18). These isolates comprised 62 distinct strains, defined on the basis of phylogenetic clustering, using cutoffs of ∼500 single-nucleotide variants (SNVs). Long-read (PacBio) sequencing was carried out on a random subset of 17 isolates (18). For the work presented here, an additional six isolates underwent long-read sequencing with PacBio technology as previously described (30).

### Sequence analysis.

We used three methods to identify chromosomally integrated Tn*4401*/*bla*_KPC_. First, we used a *de novo* assembly approach. Illumina reads from each isolate were assembled using Velvet and VelvetOptimiser (31). We then queried these with BLASTn, using 400 bp of sequence from each end of Tn*4401* in order to identify contigs containing possible Tn*4401* junction sequences. Isolates were evaluable if ≥400 bp of Tn*4401* sequence plus ≥400 bp of flanking sequence were present on a single contig for at least one of the two Tn*4401* ends. For evaluable isolates, Tn*4401* flanking sequences were first compared to known plasmid flanking sequences from previously performed long-read sequencing of a subset of the 281 isolates (18). For isolates that did not show a match to any of these plasmid references, Tn*4401* flanking sequences were used as BLASTn queries against NCBI's nucleotide database, with homology to chromosomal sequences taken as evidence for a likely chromosomal location.

Second, we used a mapping approach. For each of the 281 isolates, Illumina reads were mapped to a reference consisting of a species-specific chromosome (Table 1) plus the pKPC_UVA01 Tn*4401*b-1 sequence (18). For five isolates, there was no species-specific reference available, and we used a closely related species instead (Table 1). Mapping was performed using bwa mem version 0.7.12-r1039 (32) with default parameters, and the output was filtered to remove supplementary alignments. For read pairs where one read mapped within 1 kb of either end of the Tn*4401* sequence and the other read mapped to the chromosome, the corresponding position of mapping in the chromosome was extracted from the bam file. The length of the chromosome was divided into 1-kb nonoverlapping windows, and ≥10 reads within a single window were taken as evidence for chromosomal integration of Tn*4401*.

Third, we used long-read PacBio sequencing (18, 30), fully resolving the genetic flanking structures around Tn*4401*, in order to validate the findings of putative chromosomal integration from the short-read analyses described above. In all cases where multiple isolates were identified from the same patient using the short-read approaches described above, these represented the same strain and chromosomal locus. Therefore, we performed long-read sequencing on the earliest isolate identified from each of the eight patients, with two exceptions (i.e., six isolates in total). We did not perform long-read sequencing on CAV1351, as a later isolate from the same patient (CAV1392) was previously PacBio sequenced as part of another study (18), or on CAV1518, as this isolate was demonstrated not to have a chromosomal integration of *bla*_KPC_ by further analysis of Illumina data (see Results).

For Tn*4401* relative coverage estimation, we mapped reads to a two-contig reference (Tn*4401* contig plus chromosome contig) as described above for the mapping approach. The average coverage of Tn*4401* relative to the chromosome was calculated as (number of reads mapping to Tn*4401*/length of Tn*4401* contig) × (length of chromosome contig/number of reads mapping to chromosome).

For determination of 5-bp sequences flanking Tn*4401* in ST340 isolates, reads were mapped to the Tn*4401*b-1 reference (18) using bwa mem as described above. For each read mapped to the start/end of Tn*4401*b-1 with ≥5 bp flanking the start/end position, the 5-bp region immediately adjacent to the start/end position was extracted. Five-base-pair sequences with <10 counts were excluded to account for sequencing errors. Determination of 8-bp sequences flanking the 16-kb composite region was performed similarly, except that the CAV1417 genome was used as a reference, with reads mapping to ≥30 bp of the end of the 16-kb region within this extracted, and 8-bp flanking sequences were determined. Because the IS*15*-ΔI sequence exists as multiple copies in the ST340 isolates, it is not possible to determine flanking sequences using Illumina data for this end of the 16-kb region (Fig. 1, left side). Therefore, this analysis was only performed for the other (non-IS*15*-ΔI) end (Fig. 1, right side).

Analysis of porin genes was performed using tBLASTn comparisons against the *de novo* assembly of each patient's earliest isolate, with translated *ompK35* and *ompK36* K. pneumoniae reference sequences (AJ011501 and JX291114, respectively) as queries. Reading frame disruptions were reported as likely loss-of-function mutations.

### Expression of *bla*_KPC_.

Total RNA was extracted using the RNeasy minikit (Qiagen, GmBH, Hilden, Germany) and underwent column DNase I digestion (Qiagen) as recommended by the manufacturer. cDNA was synthesized from RNA in accordance with the manufacturer's instructions (qScript cDNA supermix; Quanta Biosciences, Gaithersburg, MD). Twenty nanograms of RNA equivalent DNA was used in triplicate. Quantitative reverse transcription-PCR (qRT-PCR) was performed using SsoFast EvaGreen supermix (Bio-Rad Laboratories, Hercules, CA), template DNA, and 500 nM each primer RT-KPC-F/RT-KPC-R, K. oxytoca rpoB-F/rpob-R, or K. pneumoniae rpoB-F/rpoB-R. Relative quantification of gene expression was determined using the averaged cycle threshold (*C_T_*) values for each isolate using the Pfaffl method (33). This equation uses an expression ratio to normalize the expression levels of *bla*_KPC_ to the transcriptional level of the constitutively expressed *rpoB* gene.

### Clinical microbiology.

Enterobacteriaceae cultured from clinical (August 2007 to December 2012) and surveillance (starting in April 2009) specimens prospectively underwent carbapenemase phenotypic testing using the modified Hodge test (August 2007 to June 2008) or the indirect carbapenemase test (July 2008 to December 2012). These tests were repeated for all isolates from frozen subculture (23). The earliest available isolate with chromosomal integration from each patient underwent multiple modes of phenotypic testing, including the VITEK 2 system using a GN70 card (bioMérieux, Durham, NC), disc diffusion, Etest (bioMérieux, Durham, NC), and broth microdilution of meropenem to determine susceptibilities by following the CLSI's and manufacturers' guidelines (34).

### Clinical characteristics.

Electronic medical records were reviewed for treatment, clinical outcome, location and timing of transfer, and exposure to other patients with *bla*_KPC_-producing Enterobacteriaceae (approved by IRB 13558).

### Accession number(s).

Sequence data have been deposited in GenBank as follows: CAV1042, CP018671.1; CAV1392, CP011578.1; CAV1453, CP018356.1; CAV1217, CP018676.1; CAV1417, CP018352.1; CAV1752, CP018362.1; CAV1755, MRWY00000000.
